# Barnhöft: a hip specific 6-item questionnaire for children

**DOI:** 10.1186/s41687-017-0024-3

**Published:** 2017-12-19

**Authors:** Bengt Herngren, Margaretha Stenmarker, Karin Enskär

**Affiliations:** 1 Department of Clinical Sciences, Lund, Orthopaedics, Lund University, SE-221 00 Lund, Sweden; 2 Futurum Academy for Health and Care Jonkoping County Council, Department of Orthopaedics, Ryhov county hospital, SE-551 85 Jonkoping, Sweden; 3 Futurum Academy for Health and Care Jonkoping County Council, Department of Paediatrics, Ryhov county hospital, SE-551 85 Jonkoping, Sweden; 4 Institute for Clinicial Sciences, Department of Paediatrics, Gothenburg University, SE-405 30 Gothenburg, Sweden; 5 Department of Nursing, School of Health and Welfare, CHILD research group, Jonkoping University, P.O. Box 1026, SE-551 11 Jonkoping, Sweden

**Keywords:** Questionnaire, Children, Hip disease, Health status, Quality of life

## Abstract

**Background:**

Health-related quality of life instruments, both general and more disease specific, would ideally be included in the evaluation of outcome in paediatric orthopaedics. The aim of this study was to translate and culturally adapt an instrument measuring hip function and pain for Swedish children 8-15 years old with a hip disorder.

**Methods:**

Translation of an established questionnaire for hip disorder in children, CHOHES, was performed and called Barnhöft. Retrospective and cognitive debriefing interviews were conducted with 15 healthy children to test for the comprehensibility of the instrument. Children with slipped capital femoral epiphysis (*n* = 25) and healthy children (*n* = 35) participated in further testing through test-retest and with the comparison of answers given in a general health-related quality of life test, EQ-5D-Y (www.euroqol.org). A multi-professional expert committee supervised the process and judged the content validity.

**Results:**

The test-retest method with a weighted Cohen’s kappa showed a good stability of the instrument. The construct validity for the pain domain (1-item) in EQ-5D-Y compared to the pain domain in Barnhöft showed a Spearman’s correlation coefficient of 0.73. The degree of hip pain in Barnhöft was also compared with the item “doing usual activities” in EQ-5D-Y with a Spearman’s correlation coefficient of 0.67.

**Conclusion:**

Barnhöft could be used as a simple 6-item questionnaire to identify children with pain and/or functional limitations due to sequelae related to a hip disease in childhood.

## Background

Children with disorders affecting the hip are primarily seen by school health personnel, physiotherapists, paediatricians or paediatric orthopaedic surgeons. Limp, reduced range of motion together with pain are often found during the clinical assessment. Our knowledge about the natural history for disorders like slipped capital femoral epiphysis (SCFE), Perthe’s disease and developmental dysplasia of the hip (DDH) together with long-term outcome after treatment has increased a lot during the last 50 years even though the aetiology has remained unclear [1–3]. The evaluation of treatment in paediatric orthopaedics has usually focused on assessment of morbidity based on clinical examination and radiographic outcome. However, outcome focusing on the impact of a disease on everyday life may be as important as the clinical findings and the radiographic appearance. The concept of health-related quality of life (HRQOL) is multidimensional and the goal is to capture the individual sense of well-being including physical, psychological, social, emotional and behavioural aspects [4]. To be able to more thoroughly evaluate surgical and/or medical treatment offered to children with hip specific disorders there is a need for data with both general and more disease specific health-related quality of life instruments to include various aspects of the impact of the disease [5].

In the literature, only one hip specific questionnaire has been validated for children [6] although several scores, designed and validated for adults, have been used in publications also for the paediatric population. The Children’s Hospital Oakland Hip Evaluation Scale (CHOHES), originally developed by Aguilar et al. in Oakland, California [7], was found to be suitable for our purpose. It was primarily designed for hip function evaluation in children with sickle-cell disease with the development of avascular necrosis of the femoral head due to the disease. The score was developed based on the Harris Hip Score for adults [8]. It was validated to use for children from 8 years of age and shown to have both good reliability and validity. The questionnaire has later also been used in England, UK, for the evaluation of hip function in children with osteonecrosis of the femoral head secondary to treatment for developmental dysplasia of the hip [9].

The aim of this study was to establish an instrument in Swedish for children 8-15 years old measuring hip function and pain in children diagnosed with a hip disorder based on the work of Aguilar et al. [7]. A cultural adaptation process together with tests for reliability and validity would then be required and the instrument possible to send by mail.

## Methods

### Study participants

Healthy children (i.e. children without any known hip disorder) and children with SCFE participated in the procedure of cultural adaptation and validation. We chose 35 healthy children, 8-15 years old, for this study through organisations offering various weekly organized sport activities within the city of Jonkoping, Sweden. Both the children and at least one parent/guardian gave their informed consent to participate.

The original developer of the instrument used 26 children with a hip disorder [7]. We chose the same number of healthy children where all but one accepted to participate. Twenty-five children, 8-15 years old, with a hip disorder were included through their consecutive registration to a Swedish national quality register for children with SCFE with surgery performed during 2011 for the index hip. A majority of Swedish hospitals treat less than two children with SCFE per year [10]. Children with SCFE in Sweden have, apart from a larger proportion of overweight and obesity, no other known comorbidities compared to a normal Swedish paediatric population [10]. We therefore for practical reasons accepted to include only healthy children in the cognitive debriefing interviews.

### Psychometric instruments

*EQ-5D-Y* [11] was used as a general instrument for HRQOL. The choice of instrument was based on a clinical interview study of a non-selected Swedish paediatric population with similar age groups that would be possible to use for comparison [12]. Our aim was to send the questionnaires by mail with the intention of using a general health questionnaire that did not have a disproportionally larger number of items than the CHOHES-instrument i.e. with the consequent risk of a lower response rate. Permission was obtained from the Eurocol group (www.eurocol.org) to use the Swedish version in this study.

*EQ-5D-VAS* records the respondent’s self-rated health on a 20-cm vertical, visual analogue scale with endpoints labelled ‘the best health you can imagine’ and ‘the worst health you can imagine’ (0-100 where 100 is the best health). The EQ-5D-VAS [13] refer to the actual situation on that very day the instrument is answered. This information can be used as a quantitative measure of health as judged by the individual respondents.

The CHOHES [7] is a 100-point, 27-item questionnaire that can be divided into 3 domains: pain, function and physical examination. The pain domain (1-item) together with the hip function domain (5-items) was used in this study. The pain scale consists of a rating for each hip with a maximum of 40 points. The function domain is based on daily activities including dressing, sitting, walking and stair climbing. This portion of the scale is scored from 0 to 32 points. We had to exclude a demonstration by the child of functional ring sitting, step height and ambulation, with the intention of using the CHOHES through mailed questionnaires. Separate written child and parental instructions were distributed. CHOHES also includes a part with evaluation by physical examination however this was not used in our study.

### Procedure of translation, cultural adaptation and validation

**Fig. 1:**
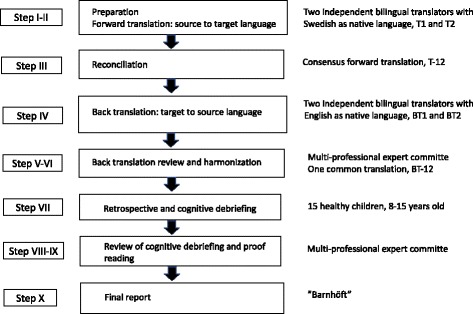
Steps for translation and cultural adaptation

The development of the Swedish version of the CHOHES, called “Barnhöft”, was performed based on the ISPOR TCA task force principles of good practice for translation and cultural adaptation for PRO [14], (Fig. 1).

*Step 1. Preparation*. Project manager together with in-country persons in close cooperation with and after approval by the original developer to utilise their instrument.

*Step 2. Forward translation*. Both translators were aware of the concepts being examined in the questionnaire and professionally had long experience working with a Swedish paediatric population.

*Step 3. Reconciliation.* This was made by the project manager, the key in-country person and both translators.

*Step 4. Back translation.* We chose a conceptual style. Both translators were blinded for the original English version of CHOHES as well as for the two different forward translations. They both had a professional experience from the medical field.

*Step 5*. *Back translation review*. Project manager together with the key in-country person based upon both the consensus version (BT12) made by the two translators and each individual back translation (BT1 and BT2).

*Step 6. Harmonization*. A multi-professional expert committee was established with a methodologist (project manager), health professionals with experience from a paediatric population, a language professional together with the translators (T1, T2, BT1 and BT2 in Fig. 1). The expert committee analysed all steps in the translation process. Decisions were made concerning semantic, idiomatic and conceptual equivalence [15]. *Face validity*, i.e. the degree to which the items of an instrument indeed looks as though they are an adequate reflection of the construct to be measured [16], was also implemented during this assessment. After consolidation of all the versions the committee developed a pre-final version of CHOHES for field testing, called the 1st version of “Barnhöft”.

*Step 7. Retrospective and cognitive debriefing.* During the test of the pre-final version we used *retrospective debriefing interviews* [17] as a qualitative method to test for understanding. This was accomplished with the assistance of 15 healthy children, 8-15 years old boys and girls with Swedish as their native language, together with two trained researchers present. After the completion of the questionnaire the interviewer checked for missing data or other problems. Then the interviewer asked if there were any items that were difficult to understand, irrelevant or offensive and if the child had any other comments to make in general. The questionnaire was adjusted accordingly and thereafter again approved by the expert committee as the 2nd version of “Barnhöft”.

*Cognitive debriefing interviews* [17] were then used with an interval of 3 months with the same group of 15 healthy children. They filled out the 2nd version of “Barnhöft”. To ensure that the meaning of the translation was equivalent to the source a debriefing process was performed individually. On an item-by-item basis each participant was asked to express the item in his or her own words which also provided interpretations for items that were problematic in translation. Emphasis was put on identification of any areas of concern in the instrument: There were no new items developed.

The questionnaire was then again adjusted accordingly.

*Step 8 and 9. Review of cognitive debriefing and proof reading.* This was made by the expert committee to assure cultural relevance. To ensure a last quality control step the original developer of the instrument participated in a meeting with the key in-country person where the whole translation and cultural adaptation process was evaluated.

*Step 10. Final report.* The expert committee established the final version of Barnhöft.

### Further testing

**Fig. 2:**
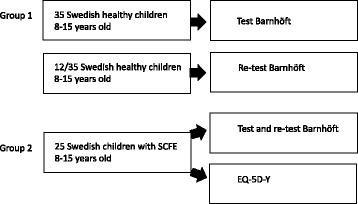
Groups for validity and reliability tests

Further testing of the final version of Barnhöft was then made with both healthy children and children with a hip disease (Fig. 2). We used a time interval of 3 months before the instrument was again presented to the children for this further testing period. In *Group one*, 10/35 were selected among the same children that were initially involved in the debriefing procedures. The reason for this selection was to utilize the positive experience of participation from the first group of children in order to minimize any difficulties to recruit another 25 healthy children. We had no children that refused to participate. For the test of the stability of the instrument, i.e. the test and re-test method, we chose 12 randomly selected children from the group of 35 healthy children.

The test and re-test method was used to evaluate the stability of the questionnaire [18] and with an equal interval of 6 weeks between the first and the second test in both groups. The intention was to keep a re-test interval of 4 weeks [19] but due to practical reasons, e.g. school vacation or abruptly postponed weekly sport activities, a majority of the healthy children (*Group one*) answered the re-test questionnaire after 6 weeks. The mailed questionnaires (*Group two*) were then distributed with the same re-test interval of 6 weeks. For those who answered the same questionnaire a second time after 6 weeks, an assumption was made that the children were in the same state regarding their hip when answering the questionnaire, a second time. This assumption was supported by the fact that 90% of children who develop bilateral SCFE do so within 18 months after the index hip was diagnosed [20] and we chose in group two to use only children with unilateral SCFE 24 months after the index hip was treated. However, we could not control whether any complications to SCFE had occurred between the first and second set of the questionnaire was distributed.

*Group one* answered the Barnhöft questionnaire with one or two researchers present to give assistance whenever necessary but they did not answer any general health-related quality of life instrument for children.

*Group two*. From a Swedish national quality register for children with a hip disease, slipped capital femoris epiphys (SCFE), an invitation was sent by mail to 26 consecutively registered children, 8-15 years old boys and girls, that had their primary surgery performed for SCFE 24 months earlier, i.e. in 2011. Only children with unilateral SCFE were invited. Data was collected through a self-administered questionnaire. The questionnaires together with information about the study were posted to the children with a return-addressed and stamped envelope. We used not more than two reminders. Information about the study was posted separately to the caretaker stating the reason for this questionnaire to be sent to their child. The caretaker was encouraged to give assistance whenever necessary but the importance of receiving the experience and opinion from the child was very much stressed. One child did not respond to this invitation and finally 25 children participated in both the test and re-test procedure. For group two we also used the Swedish version of the general child health-related quality of life instrument, EQ-5D-Y (www.euroqol.org), during the first test but not for the re-test. The reasons for this additional questionnaire (EQ-5D-Y) to be used here were to; (1) compare their answers in Barnhöft concerning pain and motion with the answers given in EQ-5D-Y and (2) compare the answers for this group with the answers from a general population of Swedish children [21].

### Psychometric properties

**Reliability** is the extent to which scores for a patient whose problems have not changed are the same for repeated measurement [16]. For test of the stability we used the test-retest method and a weighted Cohen’s kappa analysis for ordinal data [22] together with the percentage agreement method [18], a nonparametric statistical test for ordinal data.

**Validity** *.* The *content validity* - the degree to which the content of a health-related patient reported outcome (HR-PRO) instrument is an adequate reflection of the construct to be measured [16]. The relevance of items was judged by the expert committee and the comprehensibility was evaluated through cognitive interviews. *Construct validity* - the degree to which the scores of a measurement are consistent with hypotheses, e.g. relationships with scores of other instruments [16], based on the assumption that the HR-PRO instrument validly measures the construct to be measured. This was evaluated by the Spearman’s rank order correlation coefficient [23] comparing Barnhöft domains with the corresponding domains of the EQ-5D-Y questionnaire and the Multi-Dimensional Scaling (MSD) for ordinal scales was used to visualize to what extent the subparts within a domain measure the same characteristics [24]. We hypothesized that the pain domain of Barnhöft would have moderate to high (0.50 to 0.80) Spearman’s rank order correlation coefficient values with the corresponding domain of pain in EQ-5D-Y [25] and that the degree of pain in Barnhöft would correlate to the score for possibility of doing usual activities in EQ-5D-Y.

### Patient characteristics

We analysed the severity of the disease and whether a complication to the disease had occurred or not e.g. avascular necrosis.

### Data analysis

All descriptive statistical analyses were performed using SPSS Statistics for Windows (version 24.0; IBM Corp, Armonk, NY): Multi-dimensional scaling (MDS) for analysis of categorical data [24], a weighted Cohen’s kappa [22, 26] with confidence interval (CI) together with percentual agreement (PA) as described by Elisabeth Svensson for stability test of the instrument [18], and Spearman’s rank order correlation coefficient for construct validity test [23].

## Results

### General observations made during the cognitive and retrospective debriefing interviews

#### The pain domain scale

During the retrospective debriefing interviews, we found that for the children below age 11 years the term “incapacitating pain” was not so easily understood and therefore the final translation version of the questionnaire was adjusted accordingly and changed to “So much pain that I cannot even play or move around”.

#### The function domain scale

##### Dressing

The aim was to check for any problem with daily dressing that required a certain degree of flexion in the hip. In the original questionnaire, the word “discomfort” was used. We found that the corresponding most proper Swedish word was not a well-defined condition for our study population. Some children had problem with the combination of asking for “Pain, discomfort or difficulty” in the same question. They stated that pain or difficulty were hard to properly distinguish since pain itself causes difficulty. On the other hand, this did not cause them any problem to choose the best alternative for their answer but it gave them some minor initial confusion when answering the question. Therefore, we adjusted the question into “Do you have problem when putting on or taking off socks or shoes”.

##### Sitting

In the original version one item is formulated “Can sit comfortably at a table or at movies”. We revealed minor difficulties for the children below age 11 years to fully interpret the term. This part was initially adjusted already during the translation and back-translation process and the Swedish version was “Can sit comfortably at a table or in an easy chair but not on the floor”. Still, children below age 11 years had some problem to interpret this alternative properly, especially the word “comfortably”. Therefore, we further adjusted the final version and changed the first alternative to “Can sit without any problem on the floor” and the second alternative to “Can sit without any problem at a table or in an easy chair but not on the floor”.

##### Stair climbing

The term “Stair climbing: foot over foot without a railing” (or “with a railing”) was also a bit confusing to fully understand for the same age group of children below 11 years. In the Swedish version, the text was translated into “Can walk with only one foot on each step without holding (or “but must hold”) on to the staircase banisters”. A few children reported that they were unsure of their own routines and first had to go to a staircase and test themselves to be sure of which alternative they should choose.

##### Cultural adaptation of language

The term “mild pain” could not be used in Swedish since that easily could be misunderstood for something positive and therefore in the Swedish version we instead used the term “just a little pain”.

### Missing data

All questionnaires for this study were obtained without any missing data.

### Reliability

For the *stability of the instrument* the test-retest method with a weighted Cohen’s kappa with confidence interval (CI) [22] together with percentual agreement (PA) as described by Elisabeth Svensson [18] for group two (children with a hip disorder) were used, see Table 1.

**Table 1:** Stability test of Barnhöft

Item	Weighted Cohen’s kappa	Percentual agreement
Pain (v48)	0,88 (CI 0,74-1,01)	99
Dressing (v49)	0,91 (CI 0,73-1,09)	99
Walking aid (v50)	1,00 (CI 1,00-1,00)	100
Walking capacity (v52)	0,88 (CI 0,66-1,10)	96
Sitting capacity (v53)	1,00 (CI 1,00-1,00)	100
Stair climbing (v54)	1,00 (CI 1,00-1,00)	100

The 12 healthy children (group one) were too few for such a statistical evaluation. Nevertheless, the only difference registered was that 3 of 12 children chose different alternatives for walking capacity varying between “unlimited” or “long distances but limited”.

### Construct validity

This was only possible for group two since the healthy children (group one) did not answer EQ-5D-Y. Spearman’s rank order correlation coefficient was used. We compared the answers for the pain domain (1-item) in EQ-5D-Y with the pain domain in Barnhöft and found a Spearman’s rank order correlation coefficient of 0.73. It was noticed that severe pain affected the score for the function domain in Barnhöft and we therefore compared the degree of hip pain in Barnhöft with the item “doing usual activities” in EQ-5D-Y and found a Spearman’s rank order correlation coefficient of 0.67. Since the questions in the function domain of Barnhöft did not test for the same level of function as used in EQ-5D-Y it was not possible to make a comparison.

The number of children per item was not enough to make a factor analysis, a test to identify the dimensions of a test [27]. Multi-dimensional scaling (MDS) for categorical data in an ordinal scale [24, 28] was instead used to scale health-state similarity data. This methodology is based on the ranking of differences between health states combined with an associated scaling model that transforms the individual rank data into group values on the interval level. Information contained in a set of data is then represented by a set of points in a multidimensional space. We used Multi-dimensional scaling to visualize whether certain items were more closely related than others i.e. whether the different items under the domain function covered different aspects of functional capacity for the children in group two.

**Fig. 3:**
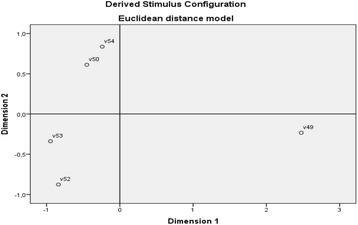
Multidimensional scaling, children with SCFE (group two)

In Table 1 the different variables used (v48-v54) are listed. We found that the capacity for stair climbing (v54) and the need for a walking aid (v50) were linked together as were also walking (v52) and sitting (v53) capacity whereas the ability to dress (v49) was found to measure a separate functional capacity (Fig. 3).

**Fig. 4:**
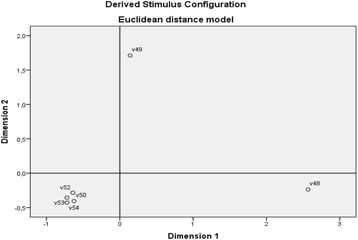
Multidimensional scaling, all children (group one and two)

Combining the healthy children (group one) with the children with a hip disorder (group two) we found that pain (v48) and the ability to dress (v49) still answered different aspects of the health status of the children whereas the different items for walking and sitting capacity tested for the same ability to move around (Fig. 4).

### Function domain (5-items)

The healthy children (group one) showed for the function domain (maximum score of 32) a median value of 32 (26-32). The children with a hip disorder (group two) had a similar median value of 32 but with a broader range (11-32). The child in group two with the lowest score had neither a severe SCFE nor a complication with avascular necrosis or chondrolysis of the femoral head but described “incapacitating” pain. The scores for this child did not change during the re-test situation.

### Pain domain (1-item)

The children in group two (children with a hip disorder) had a median score of 4 (1-5) with a lower quartile of 3.5 and an upper quartile of 5 whereas the healthy children all scored 5.

### The EQ-5D VAS

The results in group two (children with a hip disorder) showed that the children scored a mean of 88 which was equal to a Swedish general population [21].

### Group comparison EQ-5D-Y (SCFE) and general population

The Swedish EQ-5D-Y has no algorithm for evaluation other than on an item-level. We therefore chose to compare the results for EQ-5D-Y for group two (children with a hip disorder) with the 399 Swedish children from a general population published by Burström et al. [21] and their health profiles. An estimated health profile of at least ‘11122’ would then include most healthy children i.e. ‘no’ problems in the dimensions ‘mobility (walking about)’, ‘looking after myself’ and ‘doing usual activities’ and some or no problems in the dimensions ‘having pain or discomfort’ and ‘feeling worried, sad or unhappy’. This calculation showed that 375/399 (93%) of the children in the general population compared to 17/25 (68%) among children with SCFE reached this level of the health profile.

## Discussion

A hip specific questionnaire for children from age 8, Barnhöft, is now available in Swedish. The original CHOHES was used for children from age 8 years where they had a physiotherapist guiding the children through the questions while attending a re-visit in the hospital out-patient department. It is our experience that the questionnaire “Barnhöft” can be sent as a postal letter with a paper format questionnaire to the participant but with the recommendation that a parent/caretaker or a close relative should be giving assistance, especially for the children below 11 years of age.

The pain domain in Barnhöft showed a different result between healthy children (group one) and children with SCFE (group two), i.e. as one would have expected.

The children in group two (SCFE) were for the hip function domain comparable to children in the original publication by Aguilar et al. [7] with children with sickle-cell disease without any apparent avascular necrosis of the hip. We also compared children with SCFE with children with developmental dysplasia of the hip (DDH) as described by Roposch et al. [9]. We found that children with SCFE, following the score of the function domain, were comparable to children with DDH with secondary avascular necrosis grade I-II as described by Bucholtz-Ogden [29] whereas for pain the scores were comparable with grade III-IV.

We chose to follow the ISPOR task force principles of good practice for translation and cultural adaptation of PRO [14]. The need to strictly follow this sometimes costly and time-consuming methodology has been questioned [30, 31]. Epstein et al. [32] have recently stated that an expert committee is much more valuable than the procedure where back translation is included.

The original developer of the instrument [7] only included three healthy children together with 40 children with sickle-cell disease though not all of them with evidence of avascular necrosis of the hip.

In the article by Aguilar [7] both the pain and the function domain quartiles showed evidence of a ceiling effect for all children tested which was not shown when used by Roposch et al. [9] for children with avascular necrosis due to hip dysplasia. “Barnhöft” or CHOHES was never intended to be an instrument useful for healthy children so the ceiling effect seen for healthy children was expected.

When we compared the results for EQ-5D-Y with a general population (16) the calculation showed that approximately 70% of children with SCFE reached the health profile level compared to more than 90% of children in the general population. These results indicate that reduced hip function influences quality of life in the everyday life of children diagnosed with SCFE.

## Conclusion

To our knowledge, this is the only validated instrument in Swedish that assess the health status in children with a hip disorder from age 8. We would argue that the combination of a general HRQOL instrument with the 6-item hip specific instrument Barnhöft would be able to identify children with a hip disorder that have an impaired health status due to either pain, functional limitations or a combination of the two.

### Limitations

*Content validity* was not evaluated by the original developer using any qualitative analysis. The adult Harris Hip Score was their source when identifying items to be used for children. Face validity by experts was then used when the CHOHES items were finally established. In our study we made the assumption, based on epidemiological data [10], that children with SCFE were comparable to “hip-healthy” children concerning their comprehensibility of Barnhöft.

For children in *Group two* (one hip affected by SCFE) the questionnaires were distributed and answers collected by mail. In spite of written instructions, to both the child and the caretaker, we could not control for any possible caretaker bias.

*Responsiveness* – the ability of an instrument to detect change over time in the construct to be measured [16] was evaluated by the original developer but not in this study.

*Interpretability* – the degree to which one can assign clinical or commonly understood connotations to an instrument’s quantitative scores or change in scores [16] was not analysed in this study.

*Criterion validity* – the degree to which the scores of a measurement instrument constitute an adequate reflection of a gold standard [16] was not possible to evaluate due to the lack of such standard.

We used no independent measure of clinical status before the first and second presentation of the instrument to the children. We assumed that the children with SCFE who answered the test a second time (re-test) had an unchanged health status compared to when they were first exposed to the instrument. The healthy children (group one) were asked about any change in their health and functional status before presented to the re-test situation but this was unfortunately not possible to evaluate for the children in group two.

We did not recruit a completely new group of healthy children for the further testing of Barnhöft i.e. 10/35 healthy children also participated in the cognitive interviewing part of this study.

The healthy children in our study did not fill in the forms for EQ-5D-Y so we were not able to evaluate if they were giving similar answers as healthy children in a general population [21].

For the reliability test, i.e. the test of the stability of the instrument, we chose to randomly select only 12 of the 35 healthy children in Group one. This is a small sample size which might affect the results. However, in the original study only three healthy children participated together with 14 children with sickle-cell disease but without known affection of the hips [7].

For group two (children with a hip disorder) we made no analysis whether the literacy among the participants was adequate to their age [33].

**Fig. 5:**
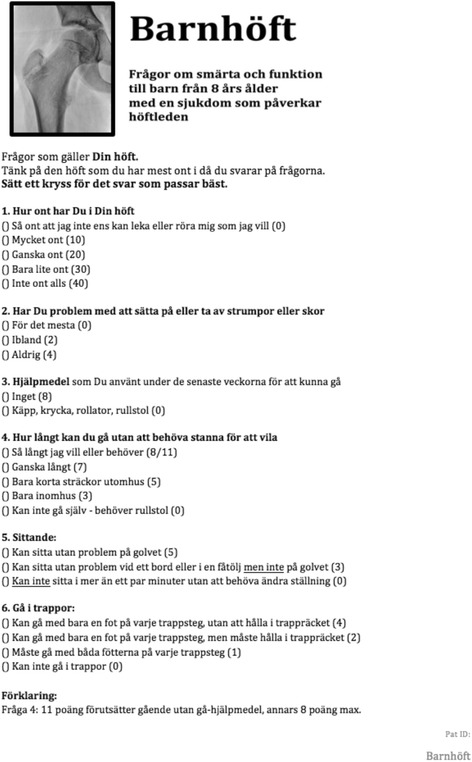
Barnhöft questionnaire

There is no other Swedish disease or hip specific questionnaire available to compare the results for “Barnhöft”. The “Barnhöft” 6-item score (Fig. 5) therefore needs to be further evaluated in a larger series of children with a hip specific disorder. to confirm the capacity to reflect the true level of pain and functional limitations for these children in relation to the severity of their disorder.
